# Magnesium and Strontium‐Doped Bioglass Nanoparticles as a Novel Formulation: Advancing Osteoconductivity and Enhancing Bone Healing Potential

**DOI:** 10.1002/smsc.70263

**Published:** 2026-03-30

**Authors:** Mehraneh Movahedi Aliabadi, Afsaneh Jahani, Ali Moradi, Nafiseh Jirofti

**Affiliations:** ^1^ Orthopedics Research Center Department of Orthopedic Surgery Mashhad University of Medical Sciences Mashhad Iran; ^2^ Bone and Joint Research laboratory Ghaem Hospital Mashhad University of Medical Sciences Mashhad Iran; ^3^ Clinical development Research Unit Ghaem hospital Faculty of Medicine Mashhad University of Medical Sciences Mashhad Iran; ^4^ Department of Biomedical Engineering Faculty of New Sciences and Technologies Semnan University Semnan Iran; ^5^ Department of Regenerative Medicine and Cell Therapy Emam Reza Hospital Mashhad University of Medical Sciences Mashhad Iran

**Keywords:** bioglass, bone regeneration, bone tissue engineering, magnesium, sol gel, strontium

## Abstract

Bioglass has gained significant attention in bone tissue engineering (BTE) due to its excellent bioactivity, osteoconductivity, and biodegradability. Recent advancements involve doping magnesium (Mg) and strontium (Sr) to enhance its osteogenic properties. This study aimed to synthesize Sr/Mg‐doped 58S bioglass nanoparticles via the sol–gel method, incorporating 58% SiO_2_, 37% CaO, 2% P_2_O_5_, 2% MgO, and 1% SrO, and to evaluate their potential for bone regeneration. The bioglass properties, including surface morphology, elemental composition, and particle size distribution, were characterized to assess their structural properties. Cell viability was evaluated over 7 days using the Resazurin assay on L929 fibroblast cells, while in vivo bone regeneration potential was assessed in rabbit skull defect models. The bioglass nanoparticles showed uniform nanoscale morphology with effective Sr and Mg incorporation. Cell viability assays demonstrated high compatibility, with no significant cytotoxicity over 7 days. In vivo results demonstrated a significant enhancement in bone formation within rabbit calvarial defects implanted with Sr/Mg‐doped bioglass (60.24 ± 2.88%) compared to the undoped bioglass group (45.80 ± 4.39%) and the control group (45.81 ± 6.01%) at 28 days post‐implantation. Sr/Mg‐doped 58S bioglass nanoparticles demonstrated enhanced biocompatibility and sustained ion release, highlighting their potential as effective nanomaterials for BTE.

## Graphical abstract

Bioactive glass nanoparticles were synthesized via the sol–gel method, incorporating osteoconductive strontium (Sr) and magnesium (Mg) ions at a novel compositional ratio to obtain a new multifunctional nanoparticle system. Comprehensive structural and morphological characterizations confirmed the successful incorporation of ions, the amorphous nature, and the nanoscale size of the particles. In vitro and in vivo evaluations demonstrated excellent biocompatibility and enhanced osteogenic potential, highlighting these Sr/Mg‐doped bioactive glass nanoparticles as a promising therapeutic candidate for bone tissue engineering applications.

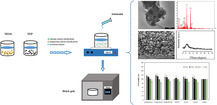

**Abbreviation**
BMP‐2: Bone morphogenetic protein‐2
BTE: Bone tissue engineering
Ca: Calcium
Cu: Copper
DLS: Dynamic light scattering
DMEM: Dulbecco's Modified Eagle's Medium
EDS: Energy‐dispersive X‐ray spectroscopy
FBS: Fetal bovine serum
FE‐SEM: Field emission scanning electron microscopy (FE‐SEM)
FTIR: Fourier‐transform infrared spectroscopy (FTIR)
HA: Hydroxyapatite
hADSCs: human adipose‐derived stem cells
ICP‐MS: Inductively coupled plasma mass spectrometry
Mg: Magnesium
Na: Sodium
OSX: Osterix
PDI: Polydispersity index
PO4: Phosphate
RUNX2: Runt‐related transcription factor 2
SBF: Simulated body fluid
Sr: Strontium
TEM: Transmission electron microscopy
TEOS: Tetraethyl orthosilicate
TEP: Triethyl phosphate
TGF‐β: Transforming growth factor‐beta
XRD: X‐ray diffraction

## 1 Introduction

A recent systematic review reported that the global prevalence of osteoporosis is as high as 18.3% [1]. Research indicates that novel bioglass shows promise as drug delivery systems for the repair or regeneration of osteoporotic bone defects [2]. Due to its excellent biological properties, including biocompatibility and bioactivity, bioglass has been widely used in the preparation of biomaterials for bone tissue engineering (BTE) [3, 4, 5]. Its capacity to integrate with mineralized bone tissue under physiological conditions, along with the adaptability offered by polymer composites, makes it highly suitable for regenerative applications such as osteoporosis treatment [6, 7]. Bioglass has shown significant potential in osteoporosis treatment by promoting osteogenesis through mechanisms such as activating Runt‐related transcription factor 2 (RUNX2) and Osterix (OSX) transcription factors in zebrafish, enhancing bone morphogenetic protein‐2 (BMP‐2) and transforming growth factor‐beta (TGF‐β) protein levels in rat models, and improving bone microstructure and screw fixation strength in the spines of sheep with osteoporosis [8, 9, 10].

Bioglass are effective in bone regeneration because they dissolve in physiological solutions such as simulated body fluid (SBF) and form a hydroxyapatite (HA)‐like layer that mimics natural bone mineral, promoting cell proliferation and hard tissue regeneration [11, 12]. Additionally, in recent years, researchers have increasingly highlighted the advantages of nano‐sized bioglass over its micro‐sized counterpart in the repair of bone defects. The smaller particle size of nano‐bioglass contributes to its enhanced surface area, improved pore volume, better cyto‐compatibility, and greater capacity to promote apatite formation—all of which are crucial for effective bone regeneration [13]. These characteristics support their role in BTE, offering innovative solutions for bone defects, trauma, and skeletal abnormalities [6, 7, 14].

58S bioglass has attracted considerable attention in recent years as a promising material for bone tissue engineering, due to its high bioactivity, osteoconductivity, and biodegradability. Its ability to promote bone regeneration and support both cell attachment and differentiation makes it especially valuable as a scaffold in regenerative applications [15, 16]. Typically, 58S bioglass consists of 58 percentage by weight (Wt%) SiO_2_, 33 wt% CaO, and 9 wt% P_2_O_5_ [17]. The relatively high silicon content plays a key role in enhancing its biocompatibility and osteoinductive behavior [16]. Among the different types of bioglasses, 58S stands out for its suitability in regenerative therapies [18, 19].

The sol–gel method, as a synthesis technique for bioglass, is conducted at substantially lower temperatures compared to conventional melt‐quenching processes, allowing for improved control over composition and structural uniformity [20]. Furthermore, bioglasses produced via the sol–gel route exhibit a larger specific surface area and enhanced porosity, which significantly increase their bioactivity and capacity for interaction with biological tissues. These attributes render sol–gel‐derived bioglasses particularly advantageous for biomedical applications [6, 21, 22]. The sol–gel method has emerged as a versatile technique for synthesizing bioglass with tunable compositions, enabling the incorporation of biologically active elements such as magnesium (Mg) and strontium (Sr) to enhance their therapeutic performance [23, 24, 25, 26, 27]. The doping of Mg into bioglass has been shown to improve bioactivity, promote cell proliferation, and enhance biocompatibility while maintaining low cytotoxicity, thereby supporting its suitability for bone regeneration applications [24]. Similarly, the doping of Sr into bioglass significantly enhances its osteogenic properties by accelerating HA formation, promoting Sr apatite deposition, and stimulating osteoblast activity, making it a valuable candidate for bone repair [25]. Recent studies have demonstrated that Sr‐doped bioglass spheres exhibit controlled ion release and the ability to enhance osteogenic differentiation in bone marrow mesenchymal stem cells from osteoporotic models, reinforcing their therapeutic potential for osteoporosis treatment [26]. Moreover, the synergistic effects of Sr doping in bioglasses have been reported to improve bioactivity, osteoblast function, and angiogenesis [27]. Additionally, Sr‐doped bioglass has been shown to suppress osteoclast differentiation and mitigate osteoporosis in animal models [28]. Given the benefits of Mg and Sr, it is anticipated that a bioglass incorporating Mg and Sr—as essential trace elements in the human body—could accelerate mineralization, promote bone formation, prevent osteoporosis, and exhibit favorable biological properties.

Therefore, it is hypothesized that bioglass incorporating Mg and Sr can facilitate bone regeneration while enhancing biological properties and mechanical strength, thereby providing superior support for osteogenesis and helping to restrain osteoporosis. Bioglass incorporating Mg and Sr has been developed in previous studies [29, 30, 31]. However, this study introduces a novel composition designed to enhance osteogenic potential, biocompatibility, and angiogenesis. To this end, Mg and Sr were incorporated into the bioglass formulation to further explore its potential in bone regeneration. Specifically, this study aimed to synthesize 58S bioglass nanoparticles doped with Mg and Sr and to evaluate their properties through a comprehensive set of microstructural, compositional, structural, and surface characterization techniques, with particular emphasis on their bone regeneration potential.

## 2 Material and Methods

### 2.1 Materials

Tetraethyl orthosilicate (TEOS, Si (OCH_2_CH_3_)4, 99%), triethyl phosphate (TEP, distilled water, nitric acid (HNO_3_, 65%)), calcium nitrate tetrahydrate (Ca (NO_3_)_2_·4H_2_O, 99%), magnesium nitrate hexahydrate (Mg (NO_3_)_2_·6H_2_O, 98%), and anhydrous strontium nitrate (Sr(NO_3_)_2_, 99.99%) were obtained from Sigma Aldrich, USA. Ethanol (CH_3_CH_2_OH, 100%) and ammonia (NH_3_, 25%) were obtained from Merck 95, Germany. For the cell study, Dulbecco's Modified Eagle's Medium (DMEM), fetal bovine serum (FBS), and Alamar Blue (Resazurin) were purchased from Gibco, USA. All materials and chemicals were used as received without further purification.

### 2.2 Bioglass Nanoparticles Synthesis

Bioglass nanoparticles based on the compositions 58% SiO_2_ – 37% CaO – 5% P_2_O_5_ (58S) and a multi‐component system 58% SiO_2_ – 37% CaO – 2% P_2_O_5_ – 2% MgO – 1% SrO (Sr/Mg‐doped 58S) were synthesized via a sol–gel method. For the preparation of Sr/Mg‐58S bioglass nanoparticles, a procedure similar to that for 58S bioglass was followed. Initially, TEOS, distilled water, 2 M nitric acid, and ethanol were combined. Subsequently, 1.13 mL of TEP and 15.72 g of calcium nitrate tetrahydrate were added to the sol, and the mixture was stirred for an additional hour to ensure homogeneity. The sol was then subjected to ultrasonic treatment, after which a 2 M ammonia solution was added dropwise as a catalyst to induce gelation and obtain a homogeneous gel. The resulting gel was dried in an oven at 70–100°C for 24 h, followed by calcination in an electric furnace at 700°C with a heating rate of 5°C per hour. In this following, for the preparation of Sr/Mg‐58S bioglass nanoparticles, a procedure similar to that of 58S bioglass was followed. Initially, TEOS, distilled water, 2 M nitric acid, and ethanol were combined and stirred magnetically for 1 h. Subsequently, TEP and 26.44 g of calcium nitrate tetrahydrate were added to the solution and stirred for an additional hour until dissolved. This was followed by the addition of 9.936 g of magnesium nitrate hexahydrate, with continued stirring for another hour. Then, 1.316 g of anhydrous strontium nitrate was added dropwise in multiple stages under continuous stirring, and the mixture was stirred for a further hour to ensure complete dissolution. Gelation was induced by the dropwise addition of a 2 M ammonia solution under mechanical mixer, which was stopped once gel formation occurred. The resulting gel was dried in an oven at a heating rate of 5–10°C/min to a final temperature of 120°C and maintained for 24 h. The dried gel was then calcined in an electric furnace at 600–800°C with a heating rate of 5°C/min for 24 h. Finally, the calcined material was ground into a fine powder and stored under dry conditions, protected from direct sunlight, until further use. Table 1 presents the molar percentage composition of the bioglass nanoparticles.

**TABLE 1:** Molar percentage composition of bioglass nanoparticles.

Samples	Code	Composition				
		**SiO**_**2**_	CaO	**P**_**2**_ **O**_**5**_	SrO	MgO
58S bioglass	58S	58	37	5	0	0
Sr/Mg‐doped 58S bioglass	Sr/Mg‐58S	58	37	2	1	2

### 2.3 Bioglass Nanoparticles Characterizations

#### 2.3.1 X‐Ray Diffraction

The synthesized bioglass nanoparticles were characterized to assess their crystallization behavior using X‐ray diffraction (XRD) with copper (Cu) Kα radiation (*λ* = 1.54056 Å) operated at 40 kV and 30 mA. The samples were scanned over a 2*θ* range of 10 to 80° at a scan rate of 8° per minute. The obtained diffraction patterns were analyzed to determine the phase composition and degree of crystallinity of the bioglass nanoparticles.

#### 2.3.2 Transmission Electron Microscopy

Transmission electron microscopy (TEM) was employed to examine the morphological and structural properties of the bioglass nanoparticles. Imaging was performed using a Philips CM120 (Holland) transmission electron microscope operated at an accelerating voltage of 120 kV, enabling high‐resolution visualization of the fine structural details of the un‐doping and Sr/Mg‐doped bioglass nanoparticles.

#### 2.3.3 Field‐Emission Scanning Electron Microscopy

The morphology of the synthesized bioglass nanoparticles was examined using a field‐emission scanning electron microscope (FE‐SEM, MIRA3, TESCAN). Prior to imaging, the powder samples were sputter‐coated with a thin layer of gold or gold‐palladium to enhance conductivity and improve image quality during FE‐SEM analysis.

#### 2.3.4 Energy‐Dispersive X‐Ray Analysis

Elemental analysis of the synthesized bioglass nanoparticles was performed using energy‐dispersive X‐ray spectroscopy (EDX) coupled with FE‐SEM. A small quantity of the powder was analyzed to verify the presence of the target elements.

#### 2.3.5 Dynamic Light Scattering

Dynamic light scattering (DLS) was employed to evaluate the particle size distribution and polydispersity index (PDI) of the synthesized bioglass nanoparticles. Measurements were performed using a Zetasizer Nano ZS (Malvern Instruments Ltd., UK) at controlled room temperature with a detection angle of 90°. For analysis, the samples were dispersed in deionized water, and fluctuations in scattering intensity were monitored to determine the hydrodynamic diameter of the particles

#### 2.3.6 Fourier Transform Infrared Spectroscopy

Functional groups were evaluated, and specific bonds between BG, Mg, and Sr in the synthesized powder were identified using Fourier transform infrared (FTIR) spectroscopy. FTIR analysis was conducted using an AVATAR 370 FTIR spectrometer (Nicolet, Thermo Scientific Co., USA), and the FTIR spectra were recorded within a wavelength range of 400–4000 cm^−1^.

#### 2.3.7 Inductively Coupled Plasma Spectroscopy

Inductively coupled plasma mass spectrometry (ICP‐MS) was utilized to assess the release profile of Sr and Mg ions from the synthesized bioglass nanoparticles. Quantification of Mg and Sr concentrations in SBF at multiple time points between 1 and 7 days was performed using an Agilent 7500 quadrupole ICP‐MS instrument. The results are expressed as the cumulative percentage of the released ions over time.

#### 2.3.8 Contact Angle

The contact angles of the synthesized bioglass nanoparticles were measured using a contact angle meter (KINO SL200B/K Series, USA). A 5 μL droplet of deionized water was gently deposited onto the surface of the powder tablet. At least six measurements were taken from different areas of each sample and averaged to ensure data reliability. Contact angle images were captured with a digital camera at room temperature within 3 s of water deposition.

#### 2.3.9 Cell Viability

The cytotoxicity of the synthesized bioglass nanoparticles was evaluated using the Resazurin reagent. L929 fibroblast cells and human adipose‐derived stem cells (hADSCs) were seeded in a 48‐well plate containing DMEM supplemented with 10% FBS and 1% penicillin‐streptomycin, and incubated for 24 h at 37°C in a humidified atmosphere with 5% CO_2_. Subsequently, different concentrations (25, 50, and 100 mg/mL) of Sr/Mg‐doped 58S and undoped 58S bioglass nanoparticles were added to the wells (*n* = 5), along with a control group without nanoparticles. Following treatment periods of 1, 3, 5, and 7 d, 10 µL of Resazurin reagent (10% v/v) was added to each well, and the plates were incubated for an additional 4 h under the same conditions [32, 33]. The absorbance was measured at 605 nm using an ELISA reader (BioTek, Bad Friedrichshall, and Germany). Finally, the percentage of cell viability was calculated using the following formula (Equation (1))

(1) $$Cell\;\text{viability}\;(\%)=\frac{Test\;\text{sample}\;\text{absorbance}}{\text{Control}\;\text{sample}\;\text{absorbance}}\times 100$$

#### 2.3.10 In Vivo Study

All animal procedures were performed in accordance with institutional ethical guidelines and approved by the Institutional Animal Care and Use Committee (IACUC). The study complied with the NIH Guide for the care and use of laboratory animals. For surgical procedures in rabbits, injectable anesthesia is preferred, with the selection of anesthetic agents based on the anticipated length of the operation. A commonly used anesthetic protocol consists of intramuscular injections of ketamine hydrochloride (35 mg/kg) combined with xylazine (5 mg/kg), which provides anesthesia for approximately 20–30 min. The rabbit is placed on a sterile drape, and the surgical site is disinfected with povidone‐iodine (Betadine) and sterile gauze to maintain sterile conditions. A sagittal incision is made along the midline of the skull, extending from the front to the back. After making the incision and dissecting the subcutaneous tissue, the cranial bone is exposed. Using a surgical drill, small and precise bone defects, up to 5 mm in diameter, are created while carefully avoiding injury to the underlying brain and meninges. Pre‐sterilized bioglass nanoparticle tablets are implanted into a critical‐sized cranial defect model, with one defect in each rabbit left empty to serve as a control. Following implantation, the surgical layers are closed in multiple stages to ensure proper wound healing. To evaluate bone regeneration and scaffold integration, cone beam computed tomography (CBCT) scans are conducted on postoperative days 7, 14, and 28. The percentage of bone regeneration is then determined using the following formula (Equation (2))

(2) $$\text{Bone regeneration}(\%)=(\frac{\text{Initial}\;\text{Volume}}{\text{Defect}\;\text{Volume}\;\text{Difference}})\times 100$$

#### 2.3.11 Pathological Assay

Bioglass nanoparticle tablets from both Sr/Mg‐doped and undoped 58S bioglass nanoparticles groups were implanted into rabbit skulls and retrieved on day 28 for histological evaluation. The harvested samples were fixed in 4% paraformaldehyde solution (pH 7.4) for 24 h, then dehydrated, cleared, and embedded in paraffin. Thin sections, 5 μm in thickness, were prepared, mounted on adhesive glass slides, and stained with hematoxylin and eosin (H&E) following standard protocols [34, 35]. Additionally, Masson's trichrome staining was employed to assess bone tissue formation and collagen deposition in the Sr/Mg‐doped 58S bioglass after exposure to an acidic medium for an appropriate duration. In this assay, collagen fibers stained blue, indicating ECM deposition. The stained sections were examined and imaged using a light microscope (Olympus BX3‐CBH).

#### 2.3.12 Statistical Analysis

All the results were presented as mean ± standard deviation (SD). Statistical software SPSS 22.0 was employed for data analysis. ANOVA was conducted, followed by Tuckey's Post Hoc test to compare the results among various groups. A *p*‐value less than 0.05 was deemed significant in this study.

## 3 Results

### 3.1 X‐Ray Diffraction Analysis

**FIGURE 1:**
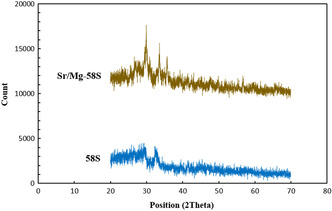
XRD analysis of Undoped (58S) and Sr/Mg‐doped (Sr/Mg‐58S) bioglass nanoparticles with different magnification.

XRD analysis was performed to characterize the phase composition of both Sr/Mg‐doped and undoped 58S bioglass nanoparticles. As shown in Figure 1, the undoped 58S bioglass exhibited a broad halo centered at 2*θ* ≈ 30°, with no sharp diffraction peaks, confirming its predominantly amorphous structure. This diffuse scattering pattern is characteristic of conventional sol–gel‐derived bioglasses, in which the rapid polycondensation process impedes long‐range atomic ordering.

In contrast, the Sr/Mg‐doped bioglass nanoparticles displayed distinct crystalline peaks superimposed on the amorphous background, particularly within the 2*θ* range of 25°–40°. Additional minor peaks at 29.9°, 33.5°, and 35.7° suggest the formation of secondary phases, potentially corresponding to strontium silicate (SrSiO_3_) or magnesium calcium silicate phases. This phenomenon is attributable to the network‐modifying effects of Sr^2+^ and Mg^2+^ ions, which promote partial crystallization upon their incorporation into the glass matrix.

Overall, XRD results confirmed that while the undoped bioglass nanoparticles remain fully amorphous, doping with Mg and/or Sr introduces a degree of crystallinity into the bioglass structure.

### 3.2 Transmission Electron Microscopy

**FIGURE 2:**
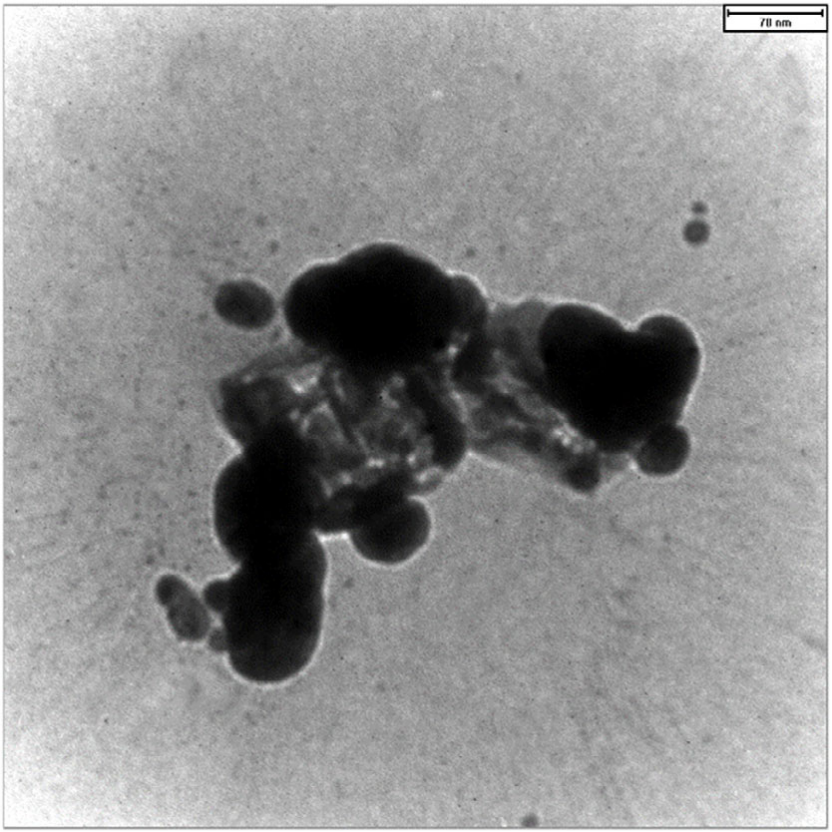
TEM images of Sr/Mg‐doped bioglass nanoparticles with different magnification.

The TEM analysis of Sr/Mg‐doped bioglass nanoparticles was conducted to characterize their morphology, size, and dispersion, as illustrated in Figure 2. The TEM images revealed that the nanoparticles predominantly exhibited a rounded shape with a relatively uniform size distribution. The particle size ranged from approximately 70–100 nm. The nanoparticles were well‐dispersed without significant agglomeration, indicating effective synthesis and stabilization methods.

### 3.3 Field Emission Scanning Electron Microscopy Morphological Analysis

**FIGURE 3:**
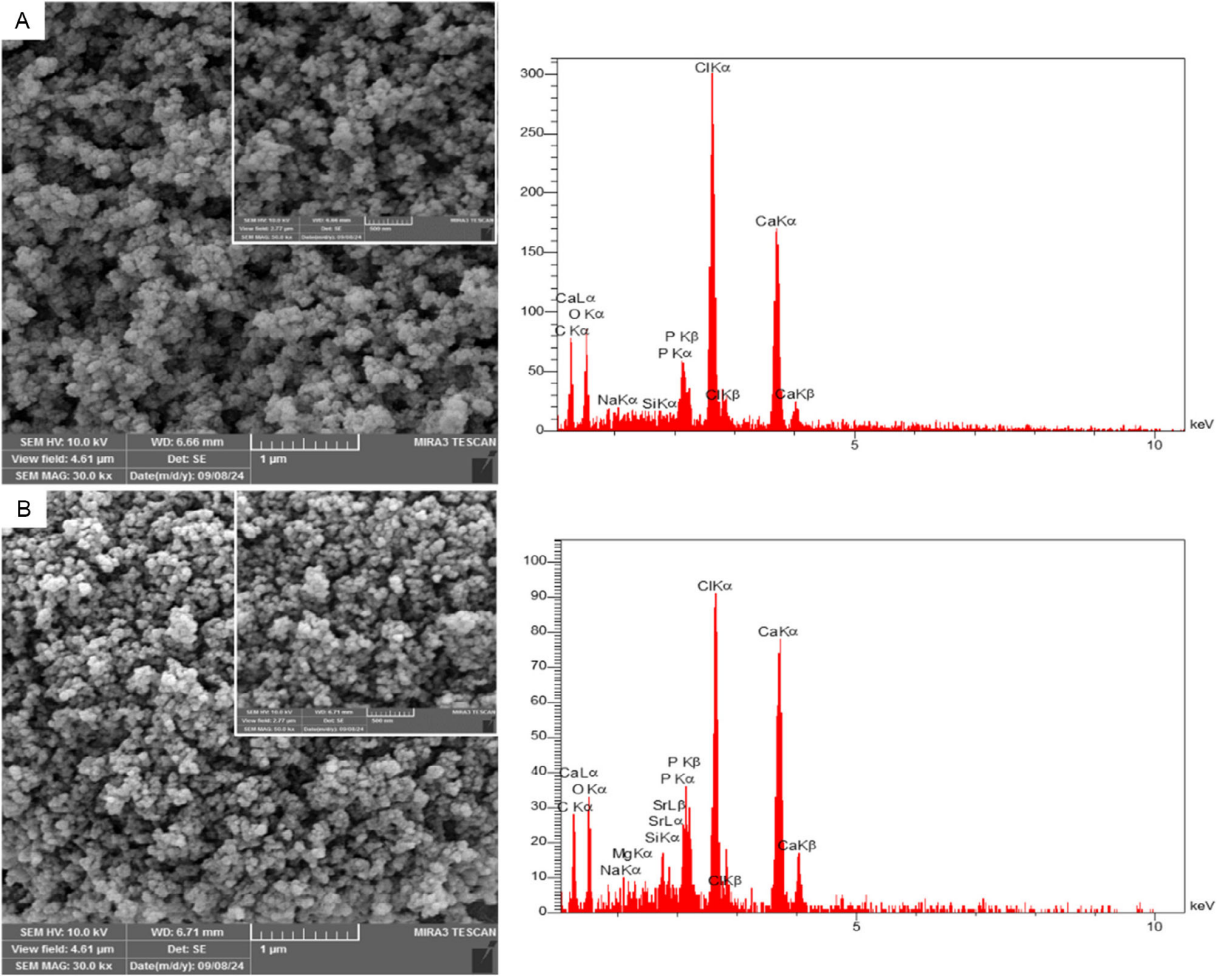
SEM images of (A) Undoped and (B) Sr/Mg‐doped bioglass nanoparticles with different magnification.

The synthesis of Mg/Sr‐doped and undoped bioglass nanoparticles was performed using the sol–gel method. The synthesized nanoparticles underwent physicochemical characterization through various analytical techniques. The surface morphology and microstructure of the synthesized bioglass powders were examined using FE‐SEM. Figure 3 illustrates the morphologies and microstructures of bioglass powders as observed by FE‐SEM. The FE‐SEM images revealed that the undoped bioglass consisted of irregular and angular particles with significant agglomeration and a broad size distribution. In contrast, the Mg/Sr‐doped bioglass exhibited more rounded and smoother particles with visibly reduced aggregation. Notably, the average particle size in both samples was below 100 nm, indicating nanoscale morphology. However, the doped sample showed smaller and more uniform particles compared to the undoped one, suggesting that the doping of Mg and Sr during synthesis led to a reduction in particle size and improved structural homogeneity. The compositional analysis via the EDX spectrum presented in Figure 3 revealed the presence of key elements, including calcium (Ca), phosphate (PO_4_), sodium (Na), Sr, and Mg in Mg/Sr‐doped BG and Ca, PO4, and Na in the undoped bioglass powder.

### 3.4 Dynamic Light Scattering Analysis

**FIGURE 4:**
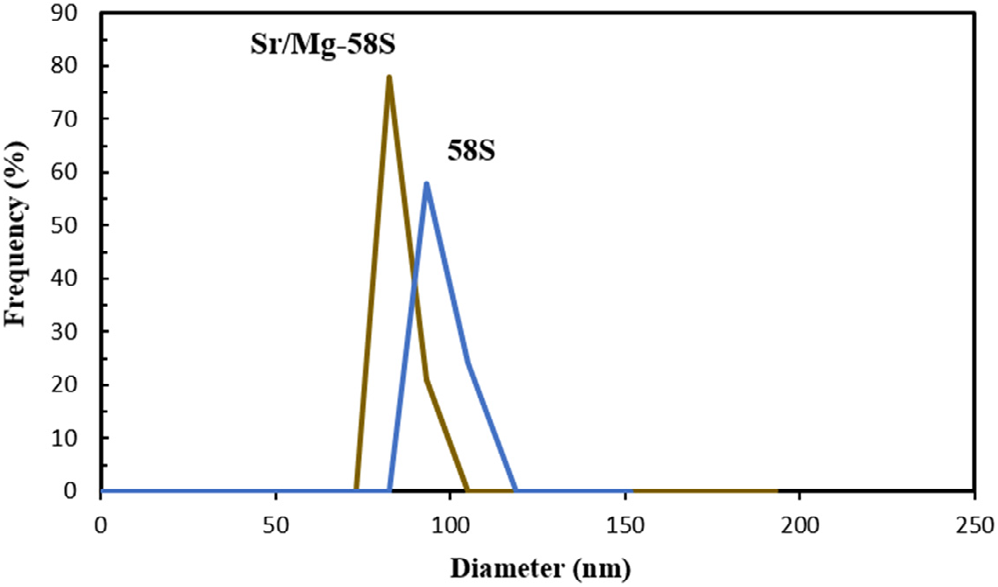
DLS Intensity‐based particle size and zeta potential distributions of undoped bioglass and Mg/Sr‐doped bioglass nanoparticles.

DLS analysis revealed that the Sr/Mg‐doped bioglass nanoparticles had a Z‐average size of 122.46 nm with a high PDI of 0.829, indicating a broad size distribution and significant heterogeneity in particle size within the sample. The undoped bioglass nanoparticles showed a larger Z‐average size of 221.79 nm with a PDI of 0.481, suggesting moderate polydispersity and the presence of larger aggregates formed during the sol–gel process. Zeta potential values for Sr/Mg‐doped bioglass nanoparticles ranged from –17.3 to –18.9 mV, indicating moderate colloidal stability. Undoped bioglass nanoparticles exhibited a wider range (–4.6 to –24.9 mV), suggesting less stable dispersion. Overall, Mg and Sr doping slightly reduced particle size and improved surface charge consistency (Table 2, Figure 4).

**TABLE 2:** Summary of DLS and zeta potential results for undoped BG and Mg/Sr‐doped bioglass nanoparticles.

Samples	Z‐Average size, nm	PDI	Size, nm
58S	221.79	0.481	108.79
Sr/Mg‐doped 58S	122.46	0.829	74.43

### 3.5 Fourier Transform Infrared Spectroscopy Analysis

**FIGURE 5:**
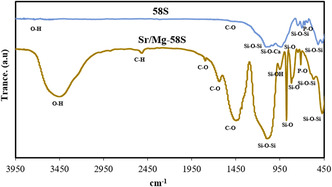
FTIR spectrums of undoped bioglass and Mg/Sr‐doped bioglass nanoparticles.

The FTIR analysis results, aimed at evaluating the functional groups of the bioglass nanoparticles, are presented in Table 3 and Figure 5. The FTIR spectrum of the 58S bioglass exhibited several characteristic absorption bands corresponding to Si—O—Si stretching and bending vibrations. A prominent band around 1050 cm^−1^ is attributed to the vibrational mode of Si—O—Ca, while the band at 920 cm^−1^ corresponds to the symmetric stretching of Si—O. A broad band observed between 2500 and 3443 cm^−1^ is assigned to C—H and O—H stretching vibrations, typically associated with surface hydroxyl groups or adsorbed water molecules. The peak at 1480 cm^−1^ is likely due to the asymmetric stretching of carbonate groups (CO_3_^2‐^). A strong absorption band at 1092 cm^−1^ corresponds to Si—O—Si asymmetric stretching, representing the silicate network of the 58S bioglass. Bands at 951 and 875 cm^−1^ may be attributed to Si—O—Si bending or P–O stretching vibrations, indicating the present of silicate and phosphate units. The absorption bands at 819 and 713 cm^−1^ are typically associated with Si–O–Si bending vibrations, while the lower frequency bands at 571 and 470 cm^−1^ correspond to bending modes of phosphate and silicate groups, respectively. The FTIR spectrum of the Sr/Mg‐doped 58S bioglass exhibited peaks between 1029 and 1103 cm^−1^, corresponding to Si—O—Si asymmetric stretching vibrations. These peaks showed shifts and broadening relative to those observed in the undoped 58S bioglass, indicating structural modifications due to doping. The band at 943 cm^−1^ is attributed to Si—OH groups or altered phosphate environments, reflecting the influence of the incorporated ions. Additionally, multiple bands observed between 747 and 636 cm^−1^ are indicative of phosphate‐related vibrations, which are likely modified by the presence of divalent cations such as Mg^2+^ and Sr^2+^. The low‐frequency bands at 517 and 469 cm^−1^ are assigned to Si—O—Si bending vibrations, although their intensities and positions differ from those in the undoped 58S bioglass.

**TABLE 3:** Comparative FTIR spectral features of undoped and Mg/Sr‐doped 58S bioglass nanoparticles.

Wavenumber, cm^−1^	Vibrational assignment	Undoped 58S bioglass	Sr/Mg‐doped 58S bioglass
3443–2500	O—H and C—H stretching (surface hydroxyl groups, adsorbed H_2_O)	Broad absorption band observed	Broad absorption band retained, indicating retained hydroxyl content
∼1480	Asymmetric stretching of carbonate groups (CO_3_^2‐^)	Distinct peak attributed to carbonate presence	Not prominently observed, possibly diminished due to doping effects
∼1092	Asymmetric stretching of Si–O–Si bonds (silicate network)	Strong, sharp absorption peak	Broadened and slightly shifted to 1029–1103 cm^−1^
∼1050	Si—O—Ca vibrational mode	Well‐defined absorption band	Overlapping with broader Si—O—Si region due to structural perturbation
∼951	Si—O—Si bending/P—O stretching	Moderate intensity band present	Possibly shifted or broadened, not individually resolved
∼943	Si—OH vibrations/altered phosphate environments	Not observed	New band indicating influence of Sr^2+^/Mg^2+^ incorporation
∼920	Symmetric stretching of Si—O bonds	Present as a distinct peak	Reduced or merged due to structural reconfiguration
819, 713	Bending vibrations of Si—O—Si linkages	Clear and well‐resolved peaks	Present with potential changes in intensity
747–636	Phosphate‐related bending vibrations	Weak or unresolved in spectrum	Multiple new bands, suggesting altered phosphate environments
571	Bending mode of phosphate groups	Detected as a moderate intensity band	Modified in both intensity and position
517, 469	Si–O–Si bending modes	Broad low‐frequency bands observed	Bands appear at slightly different positions with altered intensity

### 3.6 Inductively Coupled Plasma Spectroscopy

**FIGURE 6:**
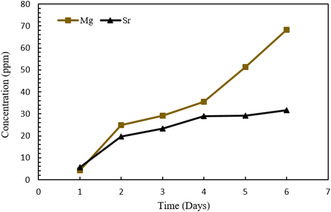
Ion release profiles of Mg/Sr‐doped bioglass nanoparticles in SBF at pH 7.4.

The release profile of Sr and Mg ions from Sr/Mg‐doped 58S bioglass nanoparticles in SBF at pH 7.4 is presented in Figure 6. The Sr ion release is fast during the 48 h of soaking in SBF at pH 7.4 it slows down and keeps increasing until 6 days of interaction showing a progressive release at physiological concentrations. This release should be beneficial for osteogenesis. The release of Mg ions from Sr/Mg‐doped 58S bioglass nanoparticles is most rapid within the first 24 h due to ion exchange and dissolution of the glass network. Sr/Mg‐doped bioglass nanoparticles released up to 24.79 ppm of Mg2+ after 48 h. The release profile indicates that Mg ion has a higher release rate compared to Sr ion over the 6‐day period. The sustained release of both Mg and Sr ions suggests the bioglass nanoparticle is undergoing continuous degradation and ion exchange in the SBF solution.

### 3.7 Contact Angle Measurement

**FIGURE 7:**
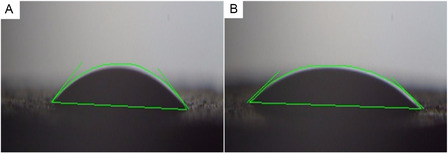
The contact angle of the (A) Undoped bioglass, and (B) Mg/Sr‐doped bioglass nanoparticles.

The hydrophilicity and wettability of bioglass nanoparticles, which are critical factors influencing cell behavior, were evaluated through contact angle measurement. Figure 7 presents the static contact angle measured at room temperature, 3 s after depositing a 5 μL water droplet onto the surface of the powder tablet. Both the undoped 58S and Sr/Mg‐doped 58S bioglass nanoparticles exhibited hydrophilic characteristics. The contact angle measured for the undoped bioglass was 26.98°, whereas the doped sample showed a lower mean contact angle of 20.55°. This decrease in contact angle indicates that the hydrophilicity of the bioglass nanoparticles increased with the incorporation of Sr and Mg ions. Furthermore, the more pronounced reduction in contact angle observed for the doped bioglass suggests accelerated droplet spreading and potentially enhanced surface reactivity or wettability, factors that may improve biological interactions.

### 3.8 Cytotoxicity Assay

**FIGURE 8:**
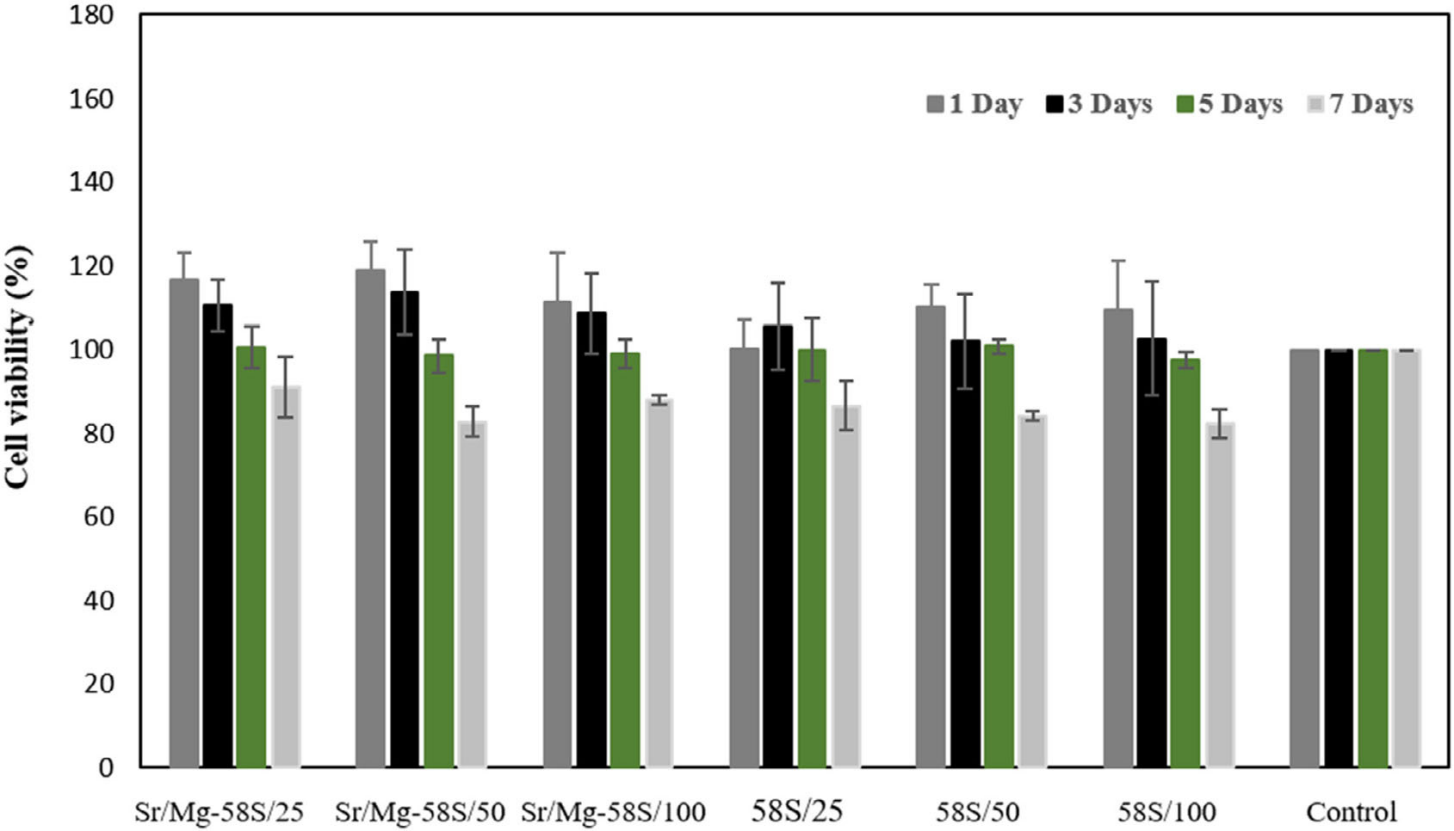
Cell Viability of L929 fibroblast cells cultured on Mg/Sr‐doped bioglass and undoped bioglass, and Control samples over 7 d. *p*‐value is defined *p* < 0.05 in compared with control sample. All results are expressed as means ± SD (Tuckey's Post Hoc test, *n* = 5).

**FIGURE 9:**
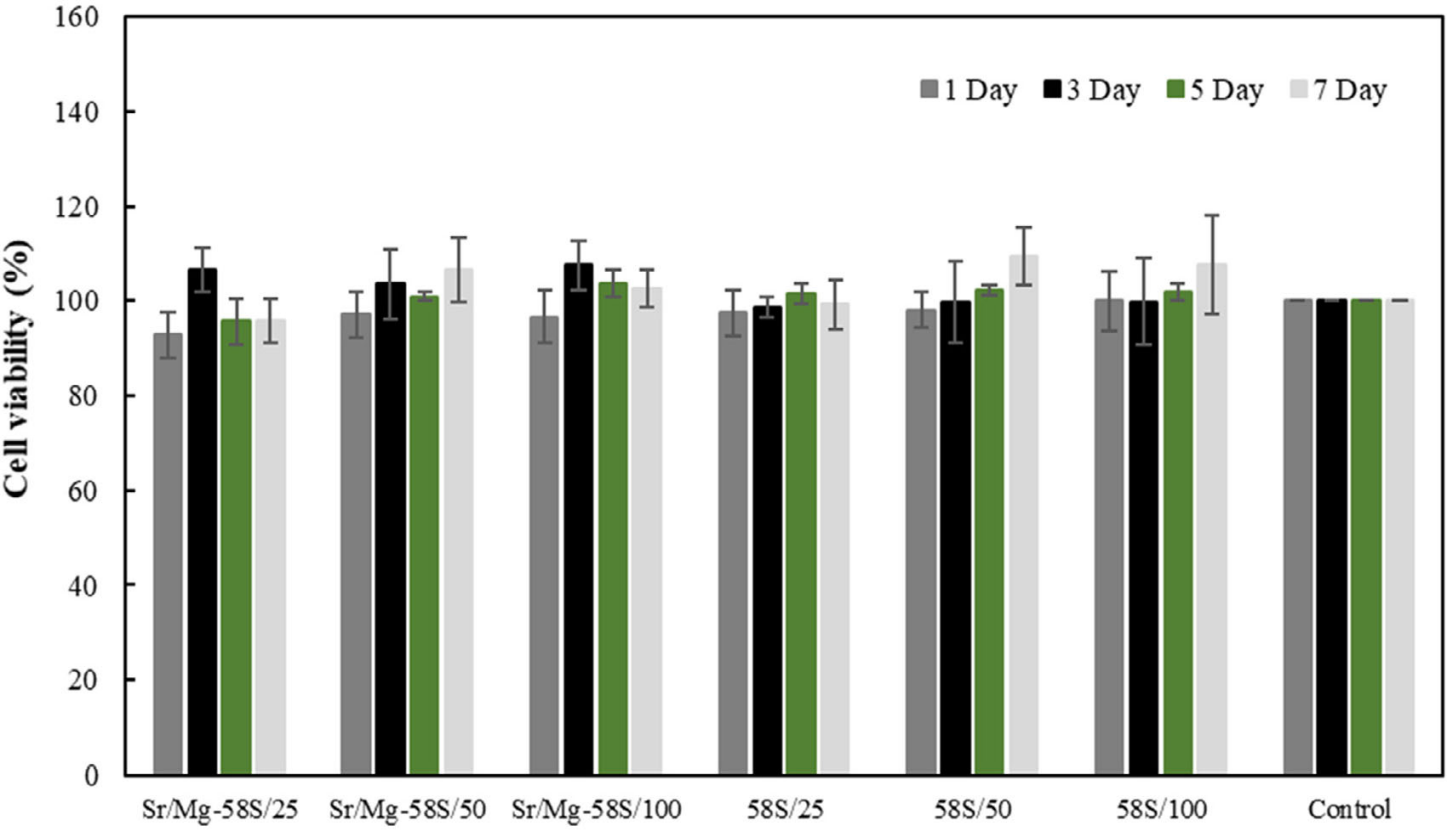
Cell Viability of hADSCs cultured on Mg/Sr‐doped bioglass and undoped bioglass, and Control samples over 7 d. *p*‐value is defined *p* < 0.05 in compared with control sample. All results are expressed as means ± SD (Tuckey's Post Hoc test, *n* = 5).

The cytotoxicity of both Mg/Sr‐doped and undoped bioglass nanoparticles was evaluated over 1, 3, 5, and 7 days using the Resazurin assay on L929 fibroblast cells, as illustrated in Figure 8. On 1 day, all bioglass nanoparticle concentrations exhibited high cell viability (>100%), with the Mg/Sr‐doped bioglass at 50 mg/ml showing the highest viability (119.10 ± 6.77%). The undoped bioglass nanoparticles also demonstrated good biocompatibility, with viability ranging from 100.30 ± 7.01% to 110.20 ± 5.22%. By 3 d, a slight decrease in cell viability was observed across all groups, however, most samples still maintained viability above 100%, indicating sustained compatibility. Notably, the Mg/Sr‐doped bioglass at 50 mg/ml recorded a viability of 113.73 ± 10.21%. On 5 d, a gradual decline in viability continued in all groups, yet most samples remained near or above 98%. The highest viability on this day was observed in the undoped bioglass at 50 mg/ml (100.94 ± 1.74%) and the Mg/Sr‐doped bioglass at the same concentration (100.62 ± 4.82%). By 7 d, further reductions in viability were noted, with the lowest value recorded for the undoped bioglass at 100 mg/ml (82.36 ± 3.49%), while the highest viability was seen in the Mg/Sr‐doped bioglass at 25 mg/ml (91.03 ± 7.24%). This decrease could be attributed to factors such as cell death, stress, or unfavorable conditions. Overall, all samples maintained viability above the 70% threshold, confirming their non‐cytotoxic nature in accordance with ISO 10 993–5 standards. In this following, the cytotoxicity of both Mg/Sr‐doped and undoped bioglass nanoparticles was assessed on hADSCs over 1, 3, 5, and 7 days, as shown in Figure 9. After 1 d, cell viability for all bioglass nanoparticle concentrations was approximately 100% or higher, comparable to the control, with the 58S bioglass at 100 mg/ml concentration exhibiting the highest viability at 100 ± 6.32%, demonstrating suitable initial cell compatibility. By 3 d, the Mg/Sr‐doped bioglass at 100 mg/ml showed an increased cell viability of 107.47 ± 5.27%, indicating possible cell proliferation stimulated by the bioglass. At 5 and 7 d, cell viability percentage generally remained at or above control levels. Overall, these findings suggest that both Sr‐Mg doped and undoped 58S bioglass nanoparticles are biocompatible.

### 3.9 In Vivo Study

**FIGURE 10:**
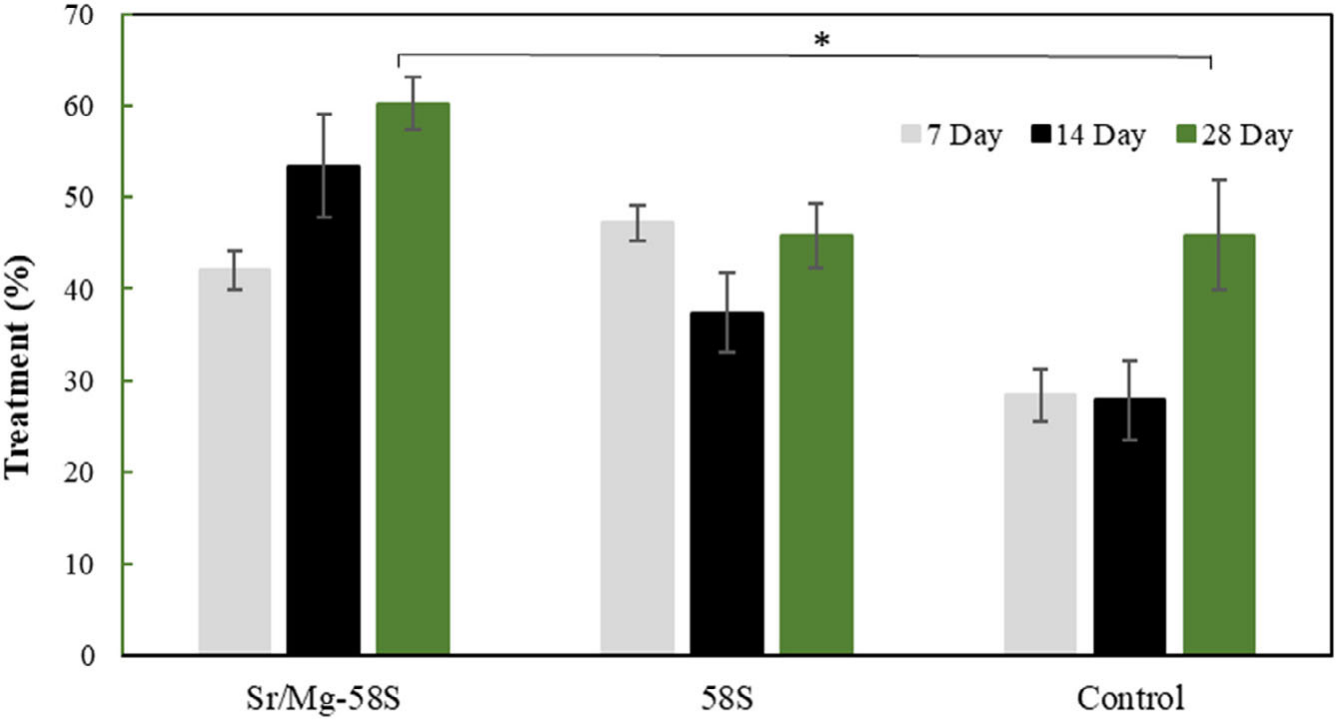
Percentage of bone healing based on changes in defect cavity volume at days 7, 14 ,and 28, relative to day 1, *p*‐value is defined *p* < 0.05 in compared with control sample.

**FIGURE 11:**
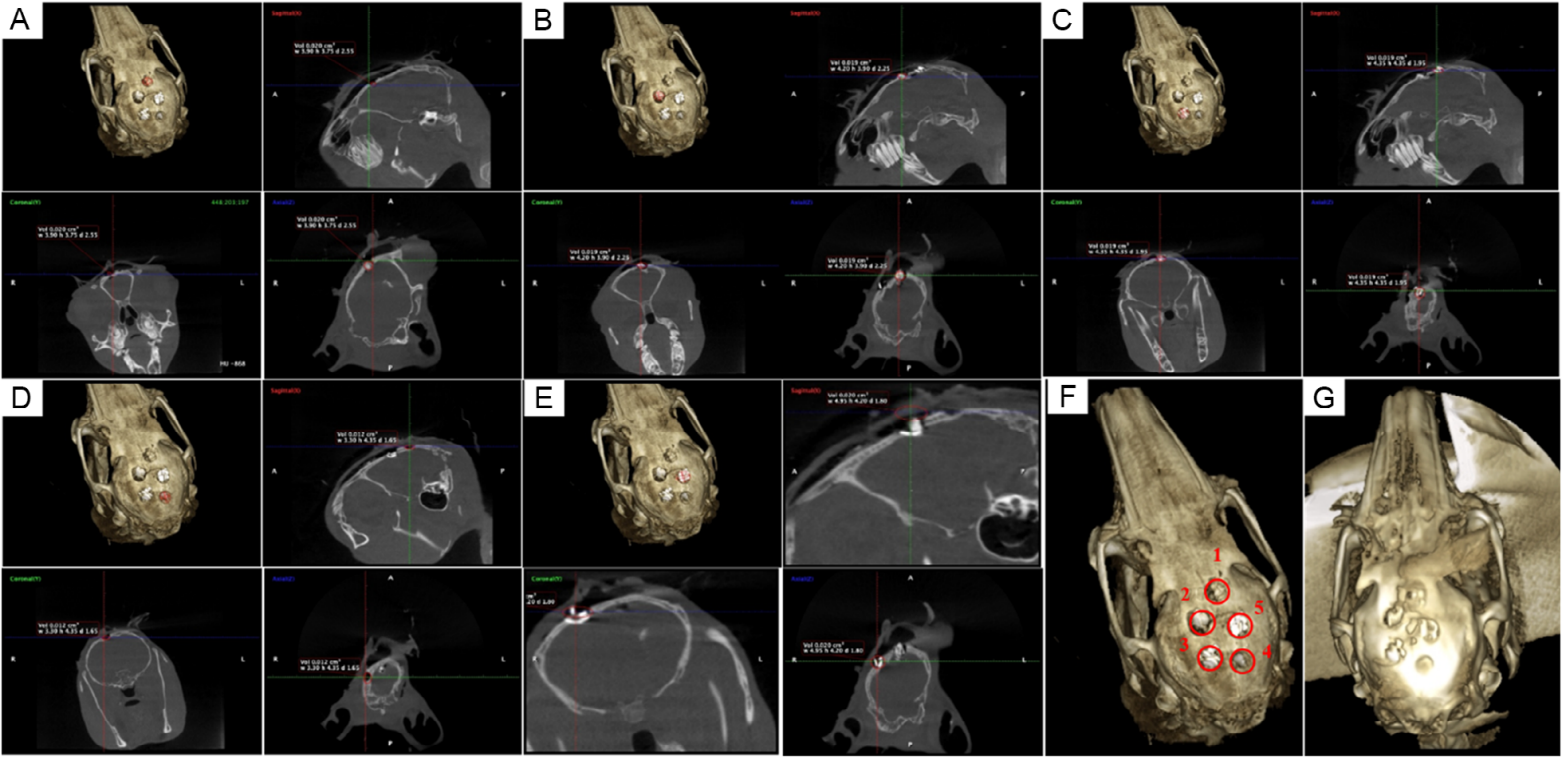
CBCT images of the cranial defect cavity at 7 and 28 d, shown in coronal, axial, and sagittal views using Romexis Viewer software: (A,B) Undoped 58S bioglass at 28 days, (C,D) Sr/Mg‐doped 58S at 28 d, (E) Untreated cavity as the control sample at 28 d, (F) Numbering of cavities and cranial defect cavity at 7 d, and (G) cranial defect cavity after treatment at 28 d post‐implantation.

A critical‐sized cranial defect model was established in rabbits to evaluate the newly bone formation capacity of Sr/Mg‐doped bioglass and undoped 58S bioglass, as illustrated by the percentage of bone healing presented in Figure 10. This assessment was conducted to quantify bone regeneration based on the reduction in defect volume following the implantation of bioglass nanoparticle tablets over various time points. Tablets composed of Sr/Mg‐doped 58S and undoped 58S bioglass were implanted into rabbit calvarial defects, while a control group remained untreated. Bone healing was evaluated at 7, 14, and 28 d post‐implantation using CBCT to determine the percentage reduction in defect volume. At 7 d, new bone formation in the Sr/Mg‐58S group reached 42.09 ± 2.12%, while the 58S group showed a slightly higher healing rate of 47.24 ± 5.63%. In contrast, the control group exhibited significantly lower healing, at 28.41 ± 2.84%. By day 14, the Sr/Mg‐58S group demonstrated an accelerated healing response, achieving 53.36 ± 1.95% bone fill, compared to 37.37 ± 4.28% in the 58S group and 27.85 ± 3.45% in the control. At the 28 d, the Sr/Mg‐58S group exhibited the highest bone healing, with 60.24 ± 2.88%, corresponding to a reduction in cavity volume to 5.57 mm^3^ correspondingly (the initial cavity volume = 13.67 mm^3^) (*p* < 0.05). This performance surpassed that of the 58S group (45.80 ± 4.39%) and the control group (45.81 ± 6.01%). These results indicate that Sr/Mg‐doped 58S bioglass significantly enhances the osteogenic potential of 58S bioglass, particularly during the early and intermediate phases of bone healing, as reflected by a more substantial reduction in defect volume over time. The coronal, axial, and sagittal of Sr/Mg‐doped 58S and undoped 58S bioglass showed in Figure 11.

### 3.10 Pathological Assay

**FIGURE 12:**
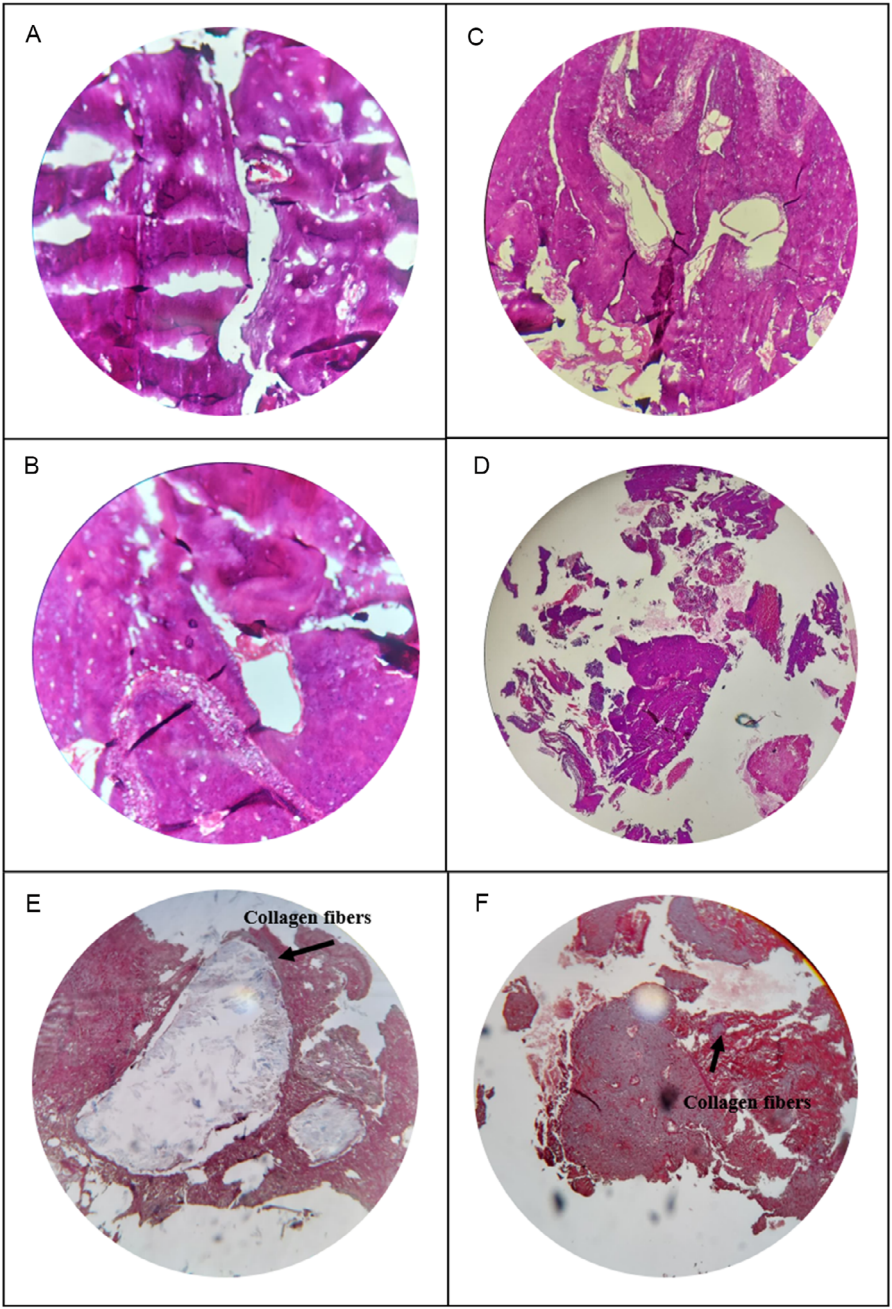
H & E staining result of (A,B) 58S bioglass, (C,D) Sr/Mg‐58S bioglass at 28 d after implantation, (E) Masson's trichrome staining results of 58S bioglass, (F) Sr/Mg‐58S bioglass at 28 d after implantation.

The pathological evaluation was conducted by assessing various healing parameters, such as inflammation, edema, granulation tissue formation, foreign body giant cells, calcification, fibrosis, and necrosis. The complete pathological data are presented in Table 4, with microscopic details of each structure illustrated in Figure 12. H&E staining indicated that neither the Sr/Mg‐58S nor the 58S bioglass samples showed the presence of adnexal structures, fungal elements, stromal reaction, or significant skin alterations. Both groups exhibited minimal edema. Necrosis was absent in the Sr/Mg‐58S group but observed in the 58S bioglass samples. Calcification was not detected in either group. Inflammation was mild in Sr/Mg‐58S and scant in 58S. Granulation tissue formation was not evident in Sr/Mg‐58S but was moderate in 58S. Foreign body giant cells were absent in both samples. Fibrosis was moderate in the Sr/Mg‐58S bioglass group but absent in the 58S. These findings suggest that the Sr/Mg‐58S bioglass induced mild inflammation and moderate fibrosis without necrosis or granulation tissue, indicating a more controlled and fibrotic tissue response. In contrast, the 58S bioglass displayed necrosis, moderate granulation tissue formation, and scant inflammation, reflecting a more active reparative response accompanied by some tissue damage. According to Figure 12, Masson's trichrome staining revealed differences in collagen fiber deposition, bone necrosis, fat necrosis, and new bone formation between the two bioglass types. The Sr/Mg‐58S sample showed moderate collagen fiber deposition (++), absence of fat and bone necrosis, and presence of new bone formation. The 58S bioglass demonstrated mild collagen fiber presence, no fat necrosis, and low new bone formation (+). Masson's trichrome staining, which highlights collagen fibers, corroborated these observations by revealing varying levels of collagen consistent with each sample. Overall, the Sr/Mg‐58S bioglass facilitated enhanced collagen deposition and bone healing with limited new bone formation, whereas the 58S bioglass induced lower collagen deposition and new bone formation, indicating distinct biological responses elicited by the two materials.

**TABLE 4:** Histopathological parameters of Mg/Sr‐doped bioglass by H & E staining.

Sample	Fibrosis	Foreign body giant cell	Granulation tissue formation	Inflammation	Calcification	Necrosis	Edema	Skin changes	Stroma reaction	Fungal element	Adnexal structure
Sr/Mg‐58S	+	–	few	mild	+	–	few	–	–	–	–
58S	Moderate	–	–	Scant	+	+	few	–	–	–	–

## 4 Discussion

Recent advancements in medicine have markedly enhanced the overall quality of life, yet the expanding elderly population introduces complex health challenges, particularly related to bone health. Age‐associated conditions such as osteoporosis, traumatic fractures, and bone loss following tumor resections significantly increase the demand for effective bone repair strategies. While autografts remain the clinical gold standard due to their osteogenic potential, their application is hindered by limited availability and donor site complications. Allografts, although more readily available, pose risks including infection and poor integration with host tissue. These limitations have driven the development of BTE approaches, with biomaterials such as calcium‐ and phosphate‐based bio ceramics gaining prominence owing to their excellent biocompatibility, osteoinductive, and osteoconductive properties. In this context, the present study focused on synthesizing Sr/Mg‐doped 58S bioglass nanoparticles via the sol–gel method to enhance their bio functionality for bone regeneration. The findings elucidate how Sr and Mg incorporation modulates the physicochemical and biological characteristics of bioglass, offering promising avenues for improving bone graft substitutes in regenerative medicine.

XRD analyses revealed that undoped 58S bioglass predominantly exhibits an amorphous structure, characteristic of sol–gel‐derived bioglasses. In contrast, doping with Sr and Mg ions induced partial crystallinity, as evidenced by the emergence of distinct diffraction peaks corresponding to potential strontium and magnesium silicate phases, consistent with findings reported in previous studies [29]. This partial crystallization can be attributed to the network‐modifying effects of the dopants during the sol–gel process, which disrupt the silicate network and promote the formation of secondary crystalline phases. Additionally, factors such as dopant interactions during calcination, lower synthesis temperatures, and extended processing times further contribute to this structural transformation that is reported in previous studies.

Morphological analysis using FE‐SEM and DLS demonstrated that doping with Sr and Mg significantly influences particle size and homogeneity The structural results demonstrated that Sr/Mg‐doped 58S bioglass notably enhanced its properties, including a more uniform nanoscale morphology, more rounded, smoother, and less agglomerated, with a smaller particles size and more uniform size distribution compared to the undoped one that confirmed this result by Li et al. [36]. TEM images confirmed that the nanoparticles predominantly exhibited a rounded morphology, with particle sizes ranging from approximately 70–100 nm. DLS data supported these observations, showing a reduced Z‐average size and improved zeta potential in doped samples, indicative of enhanced colloidal stability. Such nanoscale, homogeneous particles are desirable for biomedical applications due to their increased surface area and improved interaction with biological environments. In following, the EDX analysis confirmed the successful incorporation and even distribution of Sr and Mg in both bioglasses that confirmed this result by Bellucci et al. [37].

FTIR spectra further confirmed structural modifications, with shifts and broadening of characteristic silicate and phosphate vibrational bands, and the emergence of new peaks associated with the dopant ions However, the subtle shifts observed in our samples might indicate greater structural reorganization linked to the nanoscale morphology and sol–gel synthesis. These findings suggest that the incorporation of Sr and Mg results in more disordered and reactive glass networks, which could enhance bioactivity.

ICP analysis of ion release profiles demonstrated that both Sr and Mg ions were released in a sustained manner over a period of 7 d, with Mg exhibiting a more rapid initial release. This sustained release behavior is likely attributable to the high surface area and denser silica matrix of 58S bioglass nanoparticles. Controlled ion release is critical for stimulating osteogenic activity and facilitating bone regeneration. The progressive and sustained delivery of these bioactive ions at physiological concentrations aligns well with the design criteria for bone tissue engineering scaffolds, where gradual ion release can effectively promote cellular differentiation and matrix mineralization.

Contact angle measurements showed increased hydrophilicity in both bioglasses that Sr/Mg‐doped bioglass nanoparticles possess greater hydrophilicity than undoped samples, as indicated by a lower contact angle, potentially due to differences in surface roughness. Enhanced hydrophilicity is advantageous for cell adhesion, proliferation, and biocompatibility, suggesting that Sr/Mg‐doped bioglass may provide a more favorable surface for cell behavior.

Cytotoxicity assessment using L929 fibroblast cells and demonstrated excellent biocompatibility for both undoped and Sr/Mg‐doped bioglass nanoparticles. All samples maintained cell viability well above the ISO 10 993‐5 threshold of 70% throughout the 7 d period, with the doped samples often exhibiting slightly higher viability, particularly at lower concentrations due to its enhanced surface area. The minor decrease in viability over time, especially at higher concentrations, may be attributed to cell density‐related stress or accumulation of degradation products, but does not indicate significant cytotoxicity. The cytotoxicity assessment with hADSCs revealed that both Mg/Sr‐doped and undoped 58S bioglass nanoparticles maintained cell viability at nearly 100% or above compared to the control after 7 d, indicating their good biocompatibility with hADSCs. Notably, Mg/Sr doping enhanced cell proliferation, particularly at a concentration of 100 mg/ml after 3 d, suggesting that these nanoparticles hold promise for promoting cell growth.

The rabbit critical‐sized cranial defect model effectively demonstrated the superior osteogenic potential of Sr/Mg‐doped 58S bioglass compared to undoped 58S and untreated controls. While the 58S group initially exhibited a slightly higher bone formation at 7 d, the Sr/Mg‐doped bioglass showed significantly enhanced bone regeneration at 14 and 28  d, ultimately achieving the highest defect volume reduction (60.24 ± 2.88%) by 28 d. These findings suggest that Sr/Mg‐doped 58S bioglass synergistically enhances the early and intermediate phases of bone healing, offering a promising approach for accelerating bioglass‐based bone regeneration through more rapid defect volume reduction. This result is consistent with the findings reported by M.A. El Basiony, who demonstrated that the use of nano bioglass led to greater new bone formation compared to the control group [38]. Studies have confirmed that bioglass, owing to its calcium phosphate content and intrinsic bioactivity, can elicit specific cellular responses upon contact with native tissue, thereby establishing it as a bioactive material suitable for BTE [39, 40].

The pathological and histological evaluations revealed distinct tissue responses to Sr/Mg‐58S and 58S bioglass nanoparticles. The Sr/Mg‐58S bioglass demonstrated a more controlled healing profile, characterized by mild inflammation, moderate fibrosis, absence of necrosis and granulation tissue, and enhanced collagen fiber deposition. These features suggest a stable integration and fibrotic tissue remodeling process. In contrast, the 58S bioglass elicited a more active reparative response, marked by moderate granulation tissue formation, presence of necrosis, and mild collagen deposition, indicating some degree of tissue damage alongside healing activity. Moreover, while both materials promoted new bone formation, Sr/Mg‐58S exhibited more organized collagen architecture and absence of necrotic features, supporting its potential for safer and more regulated bone tissue regeneration.

## 5 Conclusion

In this study, Strontium (Sr)/Magnesium (Mg)‐doped 58S bioglass nanoparticles were successfully synthesized via the sol–gel method, exhibiting enhanced bioactivity, osteoconductivity, and cytocompatibility, thereby demonstrating strong potential for BTE applications. The incorporation of Sr and Mg ions significantly improved the sustained ion release, cell viability, and in vivo osteogenesis. Notably, the Sr/Mg‐doped 58S bioglass achieved the highest defect volume reduction (60.24 ± 2.88% at 28 d), indicating markedly superior and more controlled bone regeneration and tissue integration compared to undoped 58S and control samples. These results highlight the beneficial effects of Sr and Mg doping on bioglass, particularly in promoting uniform nanoscale morphology and enhanced biological performance, positioning it as a promising material for future bone regeneration applications. Nevertheless, further studies are necessary to evaluate its long‐term in vivo stability and performance.

## Author Contributions

**Mehraneh Movahedi Aliabadi**: conceptualization, data curation, writing – original draft. **Afsaneh Jahani**: conceptualization, data Curation, writing – original draft. **Ali Moradi**: supervision, resources, project administration. **Nafiseh Jirofti**: supervision, resources, funding acquisition, review & editing, project administration.

## Funding

This work is based upon research funded by Iran National Science Foundation (INSF) under project No. 4037192 and Vice‐Chancellor for Research of Mashhad University of Medical Sciences, Mashhad, Iran.

## Conflicts of Interest

The authors declare no conflicts of interest.

## Data Availability

The data that support the findings of this study are available from the corresponding author upon reasonable request.
